# In Vitro Inhibitory Mechanism Effect of TRAIP on the Function of TRAF2 Revealed by Characterization of Interaction Domains

**DOI:** 10.3390/ijms19082457

**Published:** 2018-08-20

**Authors:** Eijaz Ahmed Bhat, Chang Min Kim, Sunghwan Kim, Hyun Ho Park

**Affiliations:** 1 Department of Chemistry and Biochemistry, School of Natural Science and Graduate School of Biochemistry at Yeungnam University, Gyeongsan 38541, Korea; eijazbhat05@gmail.com; 2 College of Pharmacy, Chung-Ang University, Seoul 06974, Korea; 6427372@naver.com; 3 New Drug Development Center, Daegu-Gyeongbuk Medical Innovation Foundation, Daegu 41061, Korea; sunghwan.kim@polus-global.com; 4 R&D Center, Polus, Inc., 32 Songdogwahak-ro, Yeonsu-gu, Incheon 21983, Korea

**Keywords:** immune response, nuclear factor-κB, tumor necrosis factor-receptor associated factor, TRAF-interacting protein, protein interaction

## Abstract

TRAF-interacting protein (TRAIP), a negative regulator of TNF-induced-nuclear factor kappa-light-chain-enhancer of activated B cells (NF-κB) activation, inhibits adaptor protein TRAF2 by direct interaction and is critical in apoptosis, cell proliferation, antiviral response, and embryonic development. Although the critical function of TRAIP in NF-κB signaling is well-known, the molecular inhibitory mechanism of TRAIP remains unclear. We found that the TRAIP coiled-coil domain altered its stoichiometry between dimer and trimer in a concentration-dependent manner. Additionally, the TRAIP RING domain induced even higher-ordered assembly, which was necessary for interacting with the TRAF-N domain of TRAF2 but not TRAF1. Characterization of the TRAF-N domains of TRAF1 and TRAF2, the tentative TRAIP-binding region of TRAFs, suggested the molecular basis of the inhibitory effect of TRAIP on TRAF2 in NF-κB signaling.

## 1. Introduction

Tumor necrosis factor (TNF)-receptor associated factor (TRAF) proteins, TRAF1–TRAF7, are major signaling molecules that transduce signals for the TNF receptor (TNFR) and interleukin-1 receptor/Toll like receptor family signaling pathways in mammals and play critical roles in the regulation of the immune system and apoptosis [1,2,3]. The main function of TRAF proteins is scaffolding activity that mediates interactions between various cellular receptors, including TNFR and Toll-like receptor, and downstream effector molecules, including IRAKs, RIP1, RIP2, TAK1, BIRC2, and ASK1 [4,5,6,7]. Additionally, most TRAF proteins show E3 ubiquitin ligase activity containing an N-terminal RING domain [8], which is found in many E3 ubiquitin ligases [9]. The functions of TRAF proteins as both adaptor and E3 ubiquitin ligases are critical in inflammation, anti-viral process, and apoptosis. Because of their involvement in many human diseases, TRAF proteins have been considered important and suitable targets for therapeutic intervention [10,11].

TRAF-interacting protein (TRAIP) was initially identified as a negative regulator of TNF-induced nuclear factor (NF)-κB activation. The inhibitory function of TRAIP is mediated via a direct interaction with TRAF2 and possibly TRAF1 [12]. In addition to the main function, several novel functions of TRAIP, including proliferation, chromosome alignment at early mitotic progression, and DNA damage response, have been reported [13,14,15]. Through these multi-functional effects, TRAIP is also involved in many important cellular signaling pathways.

Although the main inhibitory function of TRAIP in TNF-induced NF-κB activation was introduced a few decades ago, the molecular mechanism of TRAIP-TRAF2 interaction-mediated inhibition of TRAF2 function is not fully understood. In this study, we found that the TRAIP coiled-coil domain forms stable dimers or trimers in solution, and the RING domain at the N-terminus of TRAIP participates in the formation of even higher-ordered oligomers. Although it has been suggested that the coiled-coil domain of TRAIP interacts with the TRAF2 coiled-coil domain (TRAF-N) to inhibit TRAF2 function [12], we found that only the coiled-coil domain of TRAIP failed to form a complex with the TRAF2 TRAF-N domain in vitro. Instead, the RING domain, which can induce higher-ordered assembly of TRAIP, was necessary for the interaction of the TRAF-N domain of TRAF2 but not that of TRAF1. Comparison of the characteristics of the TRAF-N domain of TRAF1 and TRAF2 revealed that the TRAF2 TRAF-N domain, unlike the TRAF1 TRAF-N domain, was loosely packed as a trimer in solution. This packing was important for making room for the specific interaction of TRAIP with TRAF2. We also found that the TRAF-N domain of TRAF proteins is important for trimer formation and stabilization of the TRAF domain, which is the functional unit of TRAF. Our biochemical analysis of TRAIP and TRAF1/2 provides insight into the molecular basis of TRAIP-mediated inhibition of TRAF function via a direct interaction in TNF-induced NF-κB activation.

## 2. Results and Discussion

### 2.1. TRAF-Interacting Protein (TRAIP) RING Domain Mediates Higher-Order Oligomerization of TRAIP

**Figure 1:**
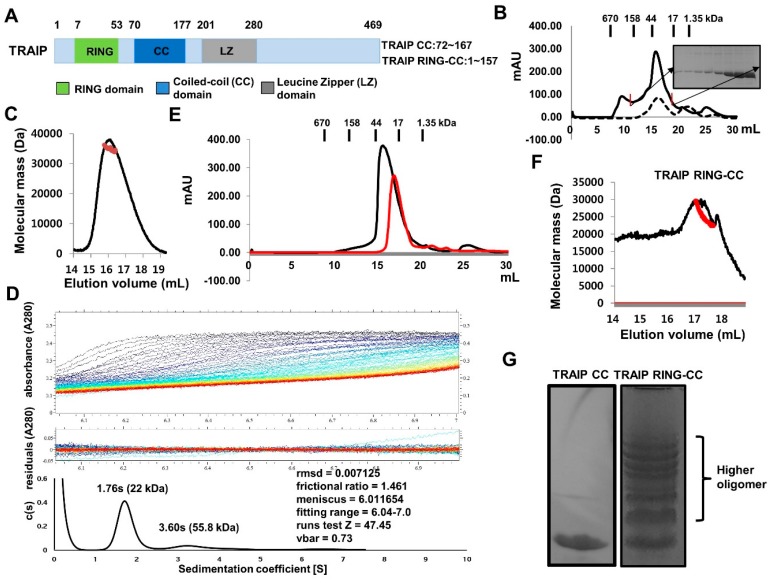
Trimeric TRAF-interacting protein (TRAIP) coiled-coil domain can form higher-order assemblies via RING domain. (**A**) The domain boundary of TRAIP. The number of amino acids of TRAIP coiled-coil (CC) and TRAIP RING-CC constructs used in this study is shown; (**B**) Comparison of size-exclusion chromatography profile between TRAIP CC (dashed-line) and TRAIP RING-CC (solid line); (**C**) Multi-angle light scattering (MALS) result for TRAIP CC. The red line indicates the experimental molecular weight; (**D**) Analytical ultracentrifugation analysis of TRAIP CC. Analytical ultra-centrifugation (AUC) experiment yielded a mass of 22 kDa (sedimentation coefficient of 1.76 s and a fractional ratio of 1.461); (**E**) Comparison of size-exclusion chromatography profile of two different concentrations of TRAIP CCs, which are a highly concentrated protein sample (Black line) and a low concentration of the protein sample (Red line); (**F**) MALS result for TRAIP RING-CC; (**G**) Native PAGE. Ladder-like bands indicating higher oligomer of TRAIP RING-CC is shown with curly brackets.

Human TRAIP (53 kDa) is composed of 469 amino acids with annotated domains including an N-terminal RING domain followed by coiled-coil (CC) and leucine zipper (LZ) domains (Figure 1A). The mouse TRAIP protein is 470 amino acids in length and shows 76% homology with human TRAIP. The RING domain has been detected in many E3 ligases and is critical for the activity of ubiquitin ligation. The E3 ligase activity of RING domain-containing TRAIP to TANK-binding kinase 1 has been reported [16]. The C-terminal region of TRAIP (residues 211–470) was found to be directly involved in the interaction with CYLD and enhanced the inhibitory activity of TRAIP [17].

For biochemical studies in vitro, we generated two expression constructs (TRAIP CC: residues 72–167 and TRAIP RING-CC: residues 1–157). Both proteins were well-expressed and purified by two quick-step chromatography processes, affinity and size-exclusion. The size-exclusion chromatography profile showed that the RING domain containing the coiled-coil domain of TRAIP (TRAIP RING-CC) was eluted from 12 to 19 mL, indicating the existence of a more highly oligomerized form, while only the coiled-coil domain of TRAIP (TRAIP CC) appeared as one single peak eluted at approximately 17 mL, indicating a homogeneous population of TRAIP CC in solution (Figure 1B). To analyze the stoichiometry of TRAIP CC in solution by calculating the absolute molecular weight, we performed multi-angle light scattering (MALS) analysis. The calculated monomeric molecular weight of TRAIP CC including the C-terminal hexa-His-tag was 12,425 Da, and the measured molecular weight based on MALS was 34,254 Da (0.9% fitting error), with a polydispersity of 1.002 (Figure 1C). Based on size-exclusion chromatography and MALS, TRAIP CC is a trimer in solution. To confirm the trimeric complex of TRAIP CC, we conducted analytical ultra-centrifugation (AUC). Interestingly, the sedimentation profile revealed that the major population of TRAIP CC in solution was centered at 1.76 s with fractional ratio of 1.46, corresponding to a dimer of approximately 22 kDa as the main peak (Figure 1D). Although the reasons for this contrasting result remain unclear, a recent our crystallographic study of TRAIP CC by our group indicated that TRAIP CC can form a stable dimer and may accommodate one more loosely packed extra coiled-coil monomer in the stably formed dimer. This characteristic of dimer-unstable trimer interconversion of TRAIP CC may explain this discrepancy. Because the use of a high concentration of the TRAIP CC sample in the MALS experiment produced the trimeric form, while a low concentration of the TRAIP CC in the AUC experiment produced a dimeric peak, this dimer-trimer interconversion may be mediated in a concentration-dependent manner. To evaluate this hypothesis, we performed size-exclusion chromatography with two different concentrations of TRAIP CC samples. As expected, the highly concentrated sample eluted earlier than the low-concentration sample, indicating that the concentration is a critical determinant of the stoichiometry of TRAIP CC (Figure 1E). The same MALS experiment was performed with TRAIP RING-CC protein sample. The calculated monomeric molecular weight of TRAIP RING-CC including the C-terminal hexa-His-tag was 18,893 Da, while the measured molecular weight by MALS was 28,536 Da (Figure 1F). The tilted line produced based on the measured data indicated that TRAIP RING-CC exists in different forms in solution (Figure 1F). To analyze the presence of the higher oligomer, we performed Native PAGE. As expected, ladder-like multi-bands were detected in the lane loaded with TRAIP RING-CC, while a single band was produced in the lane loaded with TRAIP CC (Figure 1G). This indicates that either dimeric or trimeric TRAIP can form an even higher-order homo-complex mediated by the RING-domain. 

### 2.2. Purification and Characterization of TRAF-N Domains of TRAF1 and TRAF2

**Figure 2:**
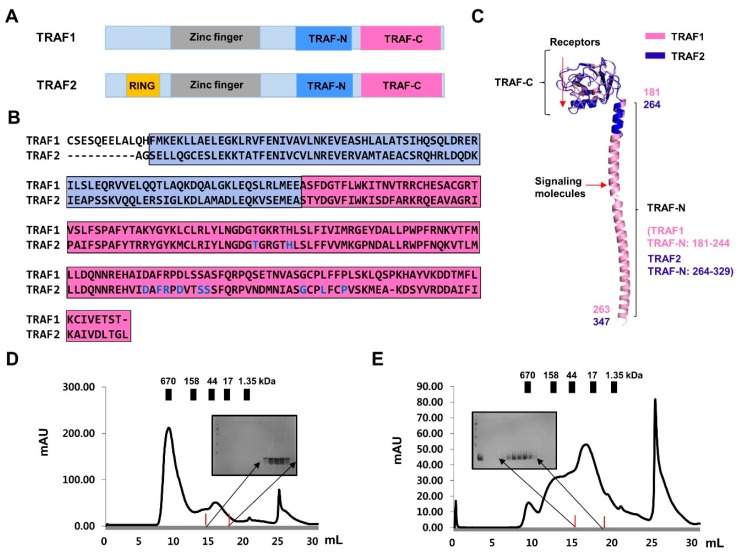
Purification of TRAF-N CC domains of TRAF1 and TRAF2. (**A**) The domain boundary of TRAF1 and TRAF2; (**B**) Sequence alignment of TRAF domains of TRAF1 and TRAF2. Blue and pink boxes indicate the TRAF-N CC and TRAF-C domains, respectively. Blue letters indicate the residues involved in the interaction with various receptors; (**C**) Ribbon diagram of the monomeric structure of TRAF1 (Pink) and TRAF2 (Blue) TRAF domain. The number of amino acids of TRAF1 TRAF-N (Pink) and TRAF2 TRAF-N (Blue) constructs used in this study are shown. D and E. Size-exclusion chromatography profiles of TRAF1 TRAF-N CC (**D**) and TRAF2 TRAF-N CC (**E**). Eluted target proteins are shown by SDS-PAGE.

The TRAF family contains a common TRAF domain at the C-terminus; six TRAF proteins, from TRAF1 to TRAF6, have been identified in mammals [2]. The C-terminally located TRAF domain can be divided into two distinct structural regions: the TRAF-N domain (a typical trimeric coiled-coil domain) and TRAF-C domain (7–8 anti-parallel β-strand folds) (Figure 2A–C). Various receptors bind to the TRAF-C domain, while various intracellular signaling molecules bind to the TRAF-N domain (Figure 2C). The TRAF domain typically forms mushroom-like trimeric structures in solution [18,19]. Despite the structural similarity of the TRAF domains, each TRAF exhibits specific biological functions with specificity to interacting upstream receptors and downstream effector molecules.

**Figure 3:**
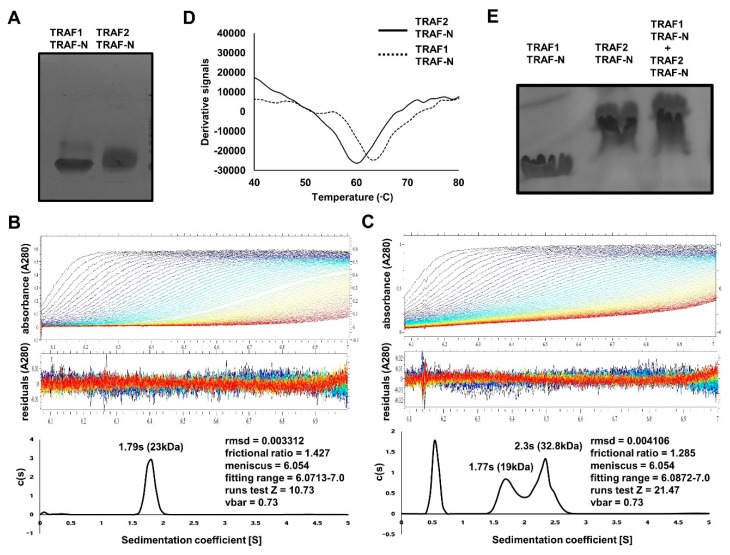
Characterization of TRAF-N domains of TRAF1 and TRAF2. (**A**) Native PAGE with purified TRAF-N domains of TRAF1 and TRAF2; (**B**) Analytical ultracentrifugation analysis of TRAF1 TRAF-N (**B**) and TRAF2 TRAF-N (**C**); (**D**) Thermostability assay; (**E**) Native PAGE of the mixture of TRAF1 TRAF-N and TRAF2 TRAF-N.

To investigate the mechanism exerted by TRAIP on TRAF using structural and biochemical methods in vitro, we purified the TRAF-N domains of TRAF1 and TRAF2, which are known to interact with TRAIP. Size-exclusion chromatography showed that both TRAF-N domains eluted at approximately 17 mL, indicating the formation of a trimer in solution (Figure 2D,E). These results agree with those of previous structural and biochemical studies [20,21]. Using purified protein samples of the TRAF-N domain of TRAF1 and TRAF2, we initially performed Native PAGE to analyze the trimeric homogeneity and compactness. Interestingly, the two TRAF-N domains showed different patterns on the Native gel, in that TRAF2 TRAF-N produced a smear, whereas TRAF1 TRAF-N produced a single clear band on the gel (Figure 3A). This indicated that trimeric TRAF2 TRAF-N is unstable and not compactly formed. The smear may have appeared because different forms were present. To confirm the homogeneity and stoichiometry of the TRAF-N domains, we performed AUC. The results showed that the two different forms (dimer and possible trimer) of TRAF2 TRAF-N were present in solution, whereas one single trimer peak was produced for TRAF1 TRAF-N, which agrees with our Native PAGE results (Figure 3B,C). Thermostability analysis showed that the homogenously populated and compact trimeric form of TRAF1 TRAF-N is more stable than TRAF2 TRAF-N, as expected (Figure 3D). Because it has been reported that dimeric TRAF2 TRAF-N interacts with one TRAF1 TRAF-N to form a stable trimeric complex [21], we analyzed the interaction between our TRAF1 TRAF-N and TRAF2 TRAF-N by Native PAGE (Figure 3E). Although a clear complex band produced by complex formation was not detected, the absence of the TRAF1 TRAF-N band strongly indicated that the TRAF-N domains of TRAF1 and TRAF2 can form a hetero-trimeric complex.

### 2.3. TRAF-N Is Critical for Functional Trimer Formation and Maintaining the Stability of the TRAF Domain

**Figure 4:**
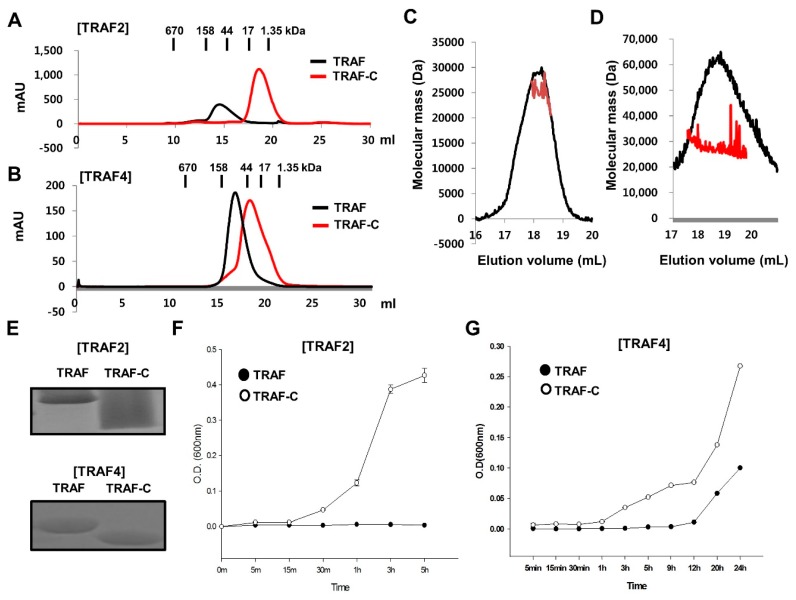
Critical effect of TRAF-N on trimer formation and stability of the TRAF domain. (**A**,**B**) Comparison of size-exclusion chromatography profile between the TRAF domain and TRAF-C domain of TRAF2 (**A**) and TRAF4 (**B**); (**C**,**D**) MALS results for TRAF2 TRAF-N (**C**) and TRAF4 TRAF-N (**D**), The red line indicates the experimental molecular weight; (**E**) Both protein samples were prepared at 1 mg/mL, Purity and concentration of initial protein samples were evaluated by SDS-PAGE; (**F**,**G**) Comparison of solubility between the TRAF2 TRAF domain and TRAF2 TRAF-C domain (**F**), and TRAF4 TRAF domain and TRAF4 TRAF-C domain (**G**) as a function of time. Turbidity caused by protein precipitation was measure at the optical density at 600 nm. Values are the means ± SD (*n* = 3).

Based on previous structural and biochemical studies of the TRAF1 TRAF domain, the TRAF-N domain is critical for trimer stabilization of TRAF1 and this trimerization is functionally important [19,20]. To further analyze the contribution of the TRAF-N domain to trimer stabilization with respect to the other TRAF family members including TRAF2, we performed various characterization assays with the TRAF domains of TRAF2 and TRAF4. Both TRAF domains of TRAF2 and TRAF4 formed trimers in solution, while the TRAF-C domain without TRAF-N domain did not (Figure 4A,B). In size-exclusion chromatography, the TRAF domains of TRAF2 and TRAF4 were eluted at approximately 14 mL, indicating that they form a trimer in solution (Figure 4A,B). However, the TRAF-C only domain, which does not contain the TRAF-N domain, was eluted at approximately 18 mL, indicating that it dissociated to a monomer in solution (Figure 4A,B). The stoichiometric changes in the TRAF domain of TRAF2 and TRAF4 by removing the TRAF-N domains were confirmed by the MALS results. The calculated molecular weights of the monomeric TRAF-C domains of TRAF1 and TRAF4 without the coiled-coil domain including the C-terminal His-tag were 26,284 and 27,269 Da, respectively, and the experimental molecular weights from MALS were 25,843 Da (0.8% fitting error), with a polydispersity of 1.000 for TRAF2 TRAF-C (Figure 4C), and 27.492 Da (1.2% fitting error), with a polydispersity of 1.000 for TRAF4 TRAF-C (Figure 4D). Based on the size-exclusion chromatography and MALS results, the TRAF-N domain is critical for the trimer assembly of TRAF2 and TRAF4, which also occurs in TRAF1 [19]. Based on current and previous observations, the TRAF-N domain is commonly necessary for forming functionally important trimers in TRAF family proteins. Because a previous study of TRAF1 showed that TRAF-N is important for TRAF domain stability and trimer formation [19], we performed stability analysis with TRAF2 and TRAF4 using two different protein samples, the TRAF domain and TRAF-C domain. After preparing the same amount of pure protein samples (Figure 4E), we performed a solubility assay. Our results showed that the monomeric TRAF TRAF-C domain was much more insoluble than the whole TRAF domain, which contains both the TRAF-N and TRAF-C domains (Figure 4F,G). These findings indicate that the TRAF-N domain is critical for trimer formation and is the functional unit of TRAF domain, and this trimerization is important for the solubility of the TRAF domain.

### 2.4. RING Domain-Mediated Oligomerization of TRAIP Is Critical for the Specific Interaction with TRAF2

**Figure 5:**
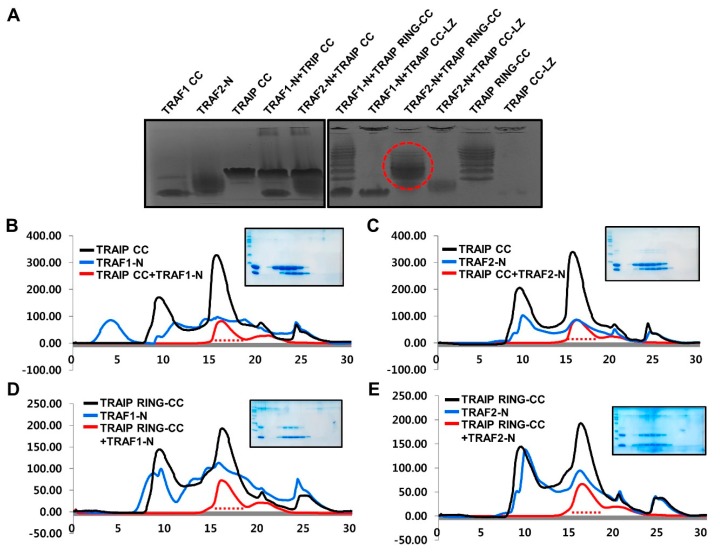
Only TRAIP RING-CC interacts with TRAF2 TRAF-N specifically in vitro. (**A**) Native PAGE shift assay with various constructs of TRAIP and TRAF-N domains of TRAF1 and TRAF2. TRAIP CC-LZ indicates TRAIP containing coiled-coil and leucine zipper domains. The red-dotted circle indicates only the shifted band that formed by formation of the complex; (**B**–**E**) Size-exclusion chromatography profiles of mixture of TRAIP CC and TRAF1 TRAF-N (**B**), TRAIP CC and TRAF2 TRAF-N (**C**), TRAIP RING-CC and TRAF1 TRAF-N (**D**), and TRAIP RING-CC and TRAF2 TRAF-N (**E**).

The interactions between the various constructs of TRAIP, which included CC, RING-CC, and CC-LZ, and TRAF-N domains of TRAF1 and TRAF2, were analyzed by Native PAGE. Although the mixtures of many combinations between TRAIP and TRAF did not produce new complex bands, the mixture of the TRAIP RING-CC and TRAF2 TRAF-N domain produced a complex band that was not detected for either TRAIP RING-CC alone or TRAF2 TRAF-N alone (Figure 5A). This result strongly indicates that only the RING domain containing the CC domain of TRAIP can interact with the loosely packed TRAF2 TRAF-N domain to inhibit TRAIP. To confirm this result, we performed size-exclusion chromatography. Although the mixtures of different combinations did not produce distinct complex peaks (Figure 5B–D) because of the similar size of trimeric complex with individual trimeric molecules, only the mixture of the TRAIP RING-CC and TRAF2 TRAF-N domain co-migrated in SDS-PAGE (Figure 5E). This result supports those of Native PAGE, showing that TRAIP RING-CC specifically interacts with TRAF2 CC in vitro. A previous yeast two hybrid study showed that TRAIP CC (corresponding amino acid 56–275) without RING domain can interact with TRAF2 and inhibit the NF-κB activation [12], which did not match with our current study. Our in vitro study with purified proteins, however, clearly showed that TRAIP-CC failed to interact to TRAF2-CC in vitro. The RING domain of TRAIP, which mediated higher oligomerization of TRAIP, was needed for interaction of TRAIP to TRAF2. Further studies for understanding this will help to reveal the inhibitory mechanism of TRAIP, which is an unanswered open question in the field of TRAF-mediated biology.

**Figure 6:**
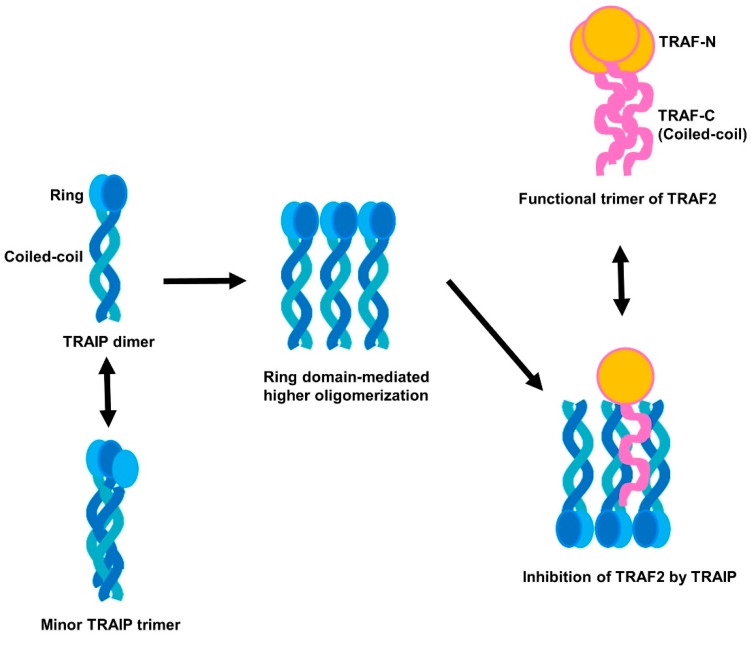
Potential inhibitory mechanism of TRAIP against TRAF2.

In conclusion, the specific interaction between TRAIP and TRAF2 was observed in vitro, and the minimal binding region of TRAIP was analyzed. The RING domain of TRAIP contributed to homo-oligomerization, which is critical for binding to the TRAF2 TRAF-N domain. This interaction strategy may be important for the inhibitory activity of TRAIP against the function of adaptor molecule TRAF. The loosely packed TRAF2 TRAF-N domain may determine the specificity of TRAIP. Collectively, based our results, TRAIP can be homo-oligomerized via the RING domain and the main form of dimeric TRAIP can hijack monomeric TRAF2 (loosely packed into trimer in certain cases) via the coiled-coil domain to inhibit assembly of the functional unit of trimeric TRAF2 during cellular signaling (Figure 6).

## 3. Materials and Methods

### 3.1. Protein Expression and Purification

To obtain C-terminally His-tagged protein samples of TRAIP, the full-length cDNA of human TRAIP (residues 1–280) was used as a template for polymerase chain reaction (PCR). Human TRAIP CC (residues 72–167), TRAIP RING-CC (residues 1–157), and TRAIP CC-LZ (residues 66–280) were amplified and cloned into the pET24a vector at the NdeI and XhoI cloning sites. The plasmid was then transformed into *Escherichia coli* BL21 (DE3) cells. The TRAF4 TRAF-C domain, which does not contain a coiled-coil domain, corresponding to amino acids 304–470 and two TRAF-N CC domains of TRAF1 (corresponding to amino acids 181–244) and TRAF2 (corresponding to amino acids 264–329) cloned into the pET24a vector were expressed in *E. coli* BL21 (DE 3) by overnight induction at 20 °C. Expressed target proteins contained a carboxyl terminal His-tag and were purified by affinity chromatography followed by size-exclusion chromatography. A superdex 200 gel filtration column 10/30 (GE Healthcare, Little Chalfont, UK) that had been pre-equilibrated with a solution of 20 mM Tri-HCl at pH 8.0 and 150 mM NaCl was used for size-exclusion chromatography. Cloning, protein expression, and purification of the TRAF domains of human TRAF2 and TRAF4 were conducted as described previously [22,23].

### 3.2. Multi-Angle Light Scattering (MALS)

The molar masses of the TRAIP CC, TRAIP RING-CC, and TRAF-C domains of TRAF2 and TRAF4 were determined by multi-angle light scattering (MALS). Purified target proteins were re-loaded onto a Superdex 200 HR 10/30 gel-filtration column (GE Healthcare) that is connected to the AKTA pure FPLC machine (GE Healthcare) and mini-DAWN treos MLAS detector (Wyatt Technology, Santa Barbara, CA, USA). The gel-filtration column had been pre-equilibrated with proper buffer.

### 3.3. Analytical Ultra-Centrifugation (AUC)

AUC data were collected at 20 °C using an XL-A analytical ultracentrifuge (Beckman Coulter, Brea, CA, USA). A concentration of 2 mg/mL of TRAIP CC for the TRAF-N domains of TRAF1 and TRAF2 in 20 mM Tris-HCl pH 8.0 and 150 mM NaCl and the reference buffer were loaded onto a dual sector Epon centerpiece (Spin Analytical, Berwick, ME, USA). Both protein samples were centrifuged at 45,000 rpm and the movements of a boundary formed by the high centrifugal force were monitored over time at a wavelength of 280 nm. The experimental data were analyzed, and c(s) was calculated using the program SEDFIT [24].

### 3.4. Native-PAGE Shift Assay

The oligomeric states of TRAIP CC and TRAIP RING-CC, as well as the complex formation of various TRAIP proteins with TRAF, were monitored by Native (non-denaturing) PAGE using a Phast System (GE Healthcare) with pre-made 8–25% acrylamide gradient gels (GE Healthcare). Purified protein samples obtained by affinity and size-exclusion chromatography were loaded onto the gel. Coomassie Brilliant Blue was used for staining.

### 3.5. Sequence Alignment

The amino acid sequences of the TRAF domains of TRAF1 and TRAF2 were analyzed using Clustal Omega (http://www.ebi.ac.uk/Tools/msa/clustalo/).

### 3.6. Solubility Assay

The assay method introduced by Bondos and Bicknell was used to analyze the solubility of target proteins [25]. Briefly, the purified TRAF domain and TRAF-C domain from size-exclusion chromatography were incubated for various times as indicated. A total of 300 µL of the 400-µL solution of each sample was used for analysis of the solubility of the target protein. The turbidity of each sample, caused by the precipitation of target protein, was measured at an optical density at 600 nm using a spectrophotometer.

### 3.7. Thermostability Assay

Protein stability was measured using the protein thermal shift starter kit (Thermo Fisher Scientific, Waltham, MA, USA). A 20-µL sample mixture of protein, thermal shift dye, and buffer was tested. The fluorescent signal was detected as a function of temperature using a CFX96 Real-Time PCR Detection System (Bio-Rad, Hercules, CA, USA). Raw data were retrieved using the Transcriptic webapp, and melt curves were generated by plotting the first derivative of the fluorescent signal as a function of temperature.

### 3.8. Complex Association Assay by Size-Exclusion Chromatography

TRAF CC and various TRAIP proteins purified from affinity chromatography were mixed in a molar ratio of approximately 1:1 and pre-incubated at 25 °C for 1 h. The mixture was then passed through a Superdex 200 gel filtration column HR 10/30 (GE Healthcare) that had been pre-equilibrated with buffer containing 20 mM Tris-HCl at pH 8.0 and 150 mM NaCl.
